# Incidence and risk factors of pressure injuries in critically ill patients with COVID-19

**DOI:** 10.1590/0034-7167-2022-0553

**Published:** 2023-12-04

**Authors:** Aline de Oliveira Ramalho, Rodrigo Augusto Gonçalves Fonseca, Eliane Mazócoli, Alessandra Marin, Paula Cristina Nogueira

**Affiliations:** I Hospital Sírio-Libanês. São Paulo, São Paulo, Brazil; II Hospital Sírio-Libanês. Brasília, Distrito Federal, Brazil; III Universidade de São Paulo. São Paulo, São Paulo, Brazil

**Keywords:** Intensive Care Units, COVID-19, Incidence, Pressure Ulcer, Nursing Care, Unidades de Cuidados Intensivos, COVID-19, Incidencia, Lesión por Presión, Atención de Enfermería, Unidades de Terapia Intensiva, COVID-19, Incidência, Lesão por Pressão, Cuidados de Enfermagem

## Abstract

**Objective:**

to analyze pressure injury (PI) incidence and risk factors in patients with COVID-19 admitted to an Intensive Care Unit and characterize the identified PIs.

**Method:**

a retrospective cohort study, consisting of 668 patients, carried out between March 2020 and February 2021. Clinical/demographic and PI variables were collected from medical records and electronic database. Data were analyzed using descriptive and inferential statistics. Logistic regression was performed to analyze risk factors for PI.

**Results:**

PI incidence was 30.2% (n=202), with the majority located in the sacral region (52.9%) and in stage 1 (39%). Risk factors were age (p<0.001), Diabetes Mellitus (p=0.005), length of stay (p<0.001), immunosuppression (p=0.034), nutritional risk (p=0.015) and mechanical ventilation (p<0.001).

**Conclusion:**

PI incidence in critically ill patients with COVID-19 was high.

## RESUMO

**Objetivo:**

analisar a incidência e os fatores de risco de lesão por pressão (LP) em pacientes com COVID-19 internados em Unidade de Terapia Intensiva e caracterizar as LP identificadas.

**Método:**

estudo de coorte retrospectivo, constituído por 668 pacientes, realizado entre março de 2020 e fevereiro de 2021. Variáveis clínicas/demográficas e das LP foram coletadas dos prontuários e banco de dados eletrônico. Os dados foram analisados por meio de estatística descritiva e inferencial. A regressão logística foi realizada para análise dos fatores de risco para LP.

**Resultados:**

a incidência de LP foi de 30,2% (n=202), sendo a maioria localizada na região sacral (52,9%) e em estágio 1 (39%). Os fatores de risco foram idade (*p*<0,001), Diabetes *Mellitus* (p=0,005), tempo de internação (p<0,001), imunossupressão (p=0,034), risco nutricional (p=0,015) e ventilação mecânica (p<0,001).

**Conclusão:**

a incidência de LP em pacientes críticos com COVID-19 foi alta.

## RESUMEN

**Objetivo:**

analizar la incidencia y factores de riesgo de lesiones por presión (LP) en pacientes con COVID-19 ingresados en Unidad de Cuidados Intensivos y caracterizar las LP identificadas.

**Método:**

estudio de cohorte retrospectivo, compuesto por 668 pacientes, realizado entre marzo de 2020 y febrero de 2021. Las variables clínico/demográficas y de LP se recogieron de historias clínicas y bases de datos electrónicas. Los datos fueron analizados mediante estadística descriptiva e inferencial. Se realizó una regresión logística para analizar los factores de riesgo de LP.

**Resultados:**

la incidencia de LP fue del 30,2% (n=202), localizándose la mayoría en la región sacra (52,9%) y en estadio 1 (39%). Los factores de riesgo fueron edad (p<0,001), Diabetes Mellitus (p=0,005), tiempo de estancia hospitalaria (p<0,001), inmunosupresión (p=0,034), riesgo nutricional (p=0,015) y ventilación mecánica (p<0,001).

**Conclusión:**

la incidencia de LP en pacientes críticos con COVID-19 fue alta.

## INTRODUCTION

According to the National Pressure Injury Advisory Panel (NPIAP), pressure injury (PI)

localized damage to the skin and/or underlying soft tissue usually over a bony prominence or related to a medical or other device. The injury can present as intact skin or an open ulcer and may be painful. The injury occurs as a result of intense and/or prolonged pressure or pressure in combination with shear^(1)^.

PIs are considered a global public health problem and have been widely discussed as an adverse event that can be avoided in most cases^(2)^. According to the National Report on Incidents Related to Health Care, PIs rank first regarding the reports made, with more than 50 thousand cases reported from September 2020 to August 2021. Of these, 6,723 refer to cases of PI stages 3 and 4, which are considered events that should never occur in health services^(3)^. In Brazil, there is a lack of robust studies that consider the national cost estimate for PI treatment. In the United States, this cost is around US$26.8 billion per year^(4)^.

It is known that patients admitted to the Intensive Care Unit (ICU) deserve greater attention with regard to PI prevention measures, as clinical, hemodynamic and mobility conditions can increase the risk of its development^(5)^. However, in mid-2020, with the arrival of the pandemic caused by SARS-CoV-2 (COVID-19), it is believed that the occurrence of these injuries may have increased, especially in critical care scenarios.

The clinical picture presented in infection by the COVID-19 virus is diverse, and varies between asymptomatic patients, others with mild involvement and non-specific signs and symptoms of acute respiratory disease. There are those who develop a severe or fatal form of the disease, characterized by pneumonia and severe respiratory impairment, conditions that make admission to the ICU indicated and necessar y^(6)^.

The pathophysiology of the virus, combined with the overcrowding of health services, the overload of care teams as well as limited access to medical and hospital products and the lack of information available to professionals in a timely manner made care for this group of patients even more complex, directly impacting health teams’ ability to prevent PI^(7)^.

Although many materials and studies have been published since the beginning of the pandemic on PI^(7,8,9,10,11)^, most of them discuss the differential diagnosis between PI and cutaneous manifestations of COVID-19, PI occurrence in prone patients and medical devices. The scientific literature still lacks robust epidemiological studies on the occurrence of these injuries in patients affected by COVID-19, especially in the Brazilian scenario. Therefore, this study sought to understand PI incidence in critically ill patients diagnosed with COVID-19 as well as analyze the risk factors found in this population, comparing those previously established in the literature, in order to determine divergences and convergences, corroborating the implementation of assertive preventive practices, according to the risk factors presented.

## OBJECTIVE

To analyze PI incidence and risk factors in patients with COVID-19 admitted to an ICU and characterize PIs in terms of stage, location and relationship with medical devices.

## METHODS

### Ethical aspects

This study was approved by the Research Ethics Committee (REC) of the hospital where it was conducted, based on Resolution 466/2012, in compliance with Brazilian National Health Council/Ministry of Health recommendations. Patients’ consent was waived, as it treated documentary research, through the collection of retrospective information, from databases and electronic medical records.

### Study design, period and place

This is a retrospective observational cohort study, guided by the recommendations contained in STrengthening the Reporting of OBservational studies in Epidemiology (STROBE) (https://www.equator-network.org/wpcontent/uploads/2015/10/STROBE_checklist_v4_combined.pdf), which was based on information recorded in the medical records of patients with COVID-19 admitted to the ICU of a large philanthropic hospital, located in the city of São Paulo, Brazil. Data collection took place between May and October 2021.

### Population and sample

The records of all patients admitted to the ICU aimed at patients diagnosed with COVID-19 were selected from March 1, 2020 to February 28, 2021. The records of patients who met the inclusion criteria were part of the sample.

### Inclusion criteria

Patients over 18 years of age, with no PI at the time of admission to ICU, with a diagnosis of COVID-19 confirmed by the RT-PCR test for COVID-19 using a nasal swab sample or tracheal secretion, admitted to the ICU until February 28, 2021 were included.

### Exclusion criteria

Patients who died or were discharged from the ICU within 24 hours of admission, with no ICU outcome until the last day of data extraction (10/31/2021), considered to be hidden and/or with restricted access to medical record information, in accordance with institutional policies, with a clinical diagnosis of COVID-19 and laboratory testing were excluded.

The total number of admissions of patients with COVID-19 to the ICU between March 2020 and February 2021 was 812, with 144 of these excluded from the study because they did not meet the inclusion criteria (84with PI present at ICU admission; 53 for length of stay <24 hours; 4 due to restriction on medical record information; 1 under the age of 18; 2 due to the absence of a PCR test for COVID-19), making up the final sample of 668 patients.

### Data collection procedures

Information was obtained through electronic medical records made by the multidisciplinary team during hospital admission in the ICU. To complement data collection with clinical information, the EPIMED® electronic cloud (information management and analysis system) was consulted, previously fed according to the sector’s routine.

The variables collected were: age; sex; Body Mass Index (BMI)^(12)^; comorbidities; Simplified Acute Physiology Score (SAPS 3) score^(13)^; pre-admission patient frailty score using the Modified Frailty Index (MFI)^(14)^; Sequential Sepsis-related Organ Failure Assessment (SOFA) ^(15)^ score (tool for assessing severity, morbidity and predicting mortality); use of sedatives; use of neuromuscular blocker; vasoactive drugs; presence of devices (nasoenteral or nasogastric cannula, indwelling bladder catheter, orotracheal cannula or tracheostomy); use of ventilatory support and length in days; classification of nutritional status on admission (by Nutritional Risk Scale (NRS) 2002)^(16)^; prone position; use of continuous renal replacement therapy (CRRT); use of extracorporeal membrane oxygenation (ECMO) and length in days; decubitus restriction; risk score for developing PI upon ICU admission using the Braden Scale^(17)^; days in the ICU until PI development; days in ICU until outcome (ICU discharge or death); hospital days admitted to the ICU; date of discharge from the ICU; and date of PI development, when applicable.

Patients who presented PI had the injury classified according to location and stage: 1, 2, 3, 4, deep tissue, unclassifiable or in mucous membrane as well as related to medical devices^(1)^. Injury stage was classified at the time of its identification and at the time of discharge from the ICU, being recorded in medical record as well as anatomical location.

It is noted that some data were not found in the medical records of all patients, such as the risk of developing PI using the Braden Scale (n= 603) and nutritional risk score using NRS-2002 (n=630).

### Analysis of results, and statistics

After collecting data from medical records and systems mentioned above, a unified database was organized in Microsoft 365 Excel® (version 2111). The PI incidence rate was calculated following NPIAP 2019 recommendations^(1)^, namely: total number of patients with PI developed in the ICU in the period, divided by the number of patients exposed to the risk of developing PI in the period, multiplied by 100.

Statistical analysis was carried out with the help of a statistical professional with experience in this study model, using the Statistical Package for the Social Sciences program (IBM SPSS classic version®). Descriptive statistics were used to characterize the sample and PI. For univariate association analysis between dependent variable (presence of PI) and demographic and clinical variables, the following statistical tests were used: Fisher’s exact and extension; Pearson’s chi-square; Student’s t for independent samples; and Mann-Whitney, depending on the variable.

Variables that presented p<0.20 (20%) in univariate analysis were concomitantly submitted to adjusted logistic regression, assessing relationships between demographic/clinical variables and the risk of developing PI. The level of statistical significance adopted in the study was 5% (p< 0.05).

## RESULTS

The study population was 668 patients, mean age 64.4 years (SD = 14.6), with a predominance of male patients (n = 518/77.5%) and white ethnicities (n = 560 /94.3%). Among the comorbidities, hypertension, Diabetes Mellitus and dyslipidemia were the most frequent, with 53.1%, 30.1% and 26.2%, respectively.

**Figure 1:**
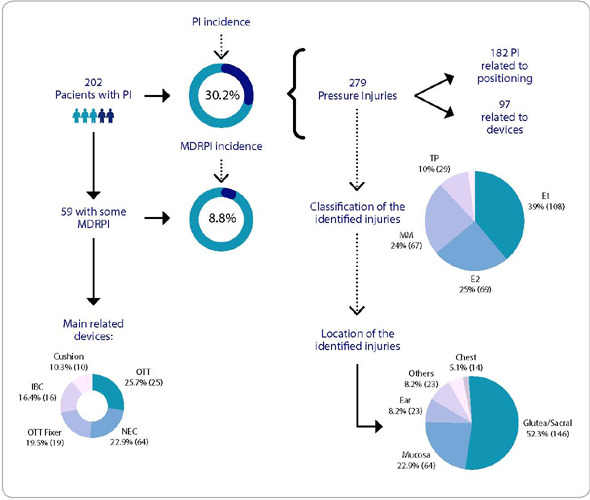
Characteristics of pressure injuries according to stages, locations and devices involved. Sao Paulo, Sao Paulo, Brazil, 2022 PI - pressure injury; MDRPI - medical device-related pressure injury; OTT - orotracheal tube; IBC - indwelling bladder catheter; NEC - nasoenteral catheter.

The average length of stay in the ICU was 13.5 days (SD=14.8), with the majority of patients being discharged as the outcome. The mean number of days spent in the ICU of patients who did not develop PI was 8.3, representing less than 1/3 of the time that patients who developed PI were admitted to hospital, with a mean of 25.6 days. PI incidence rate was 30.2% (202/668 patients). The average length between admission and PI diagnosis was 9.6 days (SD = 8.9), with the gluteal/sacral region being the main site of involvement. A patient could present more than one PI, with 279 PIs being identified, distributed across different stages. Specifically, regarding medical device-related pressure injury (MDRPI) incidence, a rate of 8.8% (59/668) was identified. The same patient could present up to 3 MDRPI, with a total number of 97 injuries, with devices related to ventilatory assistance being the main responsible for the appearance of MDRPI, followed by catheters for enteral feeding. In Figure 1, data on PI/MDRPI incidence, location and classification are presented as well as the medical devices associated with MDRPI.

In Tables 1 and 2, the risk factors for developing PI are presented according to the univariate analysis and regression analysis, respectively. In order to organize data presentation, variables were grouped in tables, such as clinical profile, screening and risk assessment tools, intensive support and hospital admission outcome.

**Table 1:** Demographic and clinical categorization of patients with and without pressure injuries. Sao Paulo, Sao Paulo, Brazil, 2022

Variable	Without PI (n: 466)	With PI (n: 202)	p value
Clinical profile			
Age in years (M-SD)	61.2 (14.6)	71.9 (11.7)	<0.001*
Sex (male)	362 (77.7%)	156 (77.2%)	0.897**
Sex (female)	104 (22.3%)	46 (22.8%)	0.897**
Ethnicity (white)	388 (93.3%)	172 (96.6%)	0.215***
Hypertension	233 (50%)	122 (60.4%)	0.013**
Diabetes Mellitus	123 (26.4%)	78 (38.6%)	0.002**
Chronic kidney failure	23 (4.9%)	18 (8.9%)	0.049**
Immunosuppression	14 (3%)	16 (7.9%)	0.005**
Cardiac insufficiency	5 (1.1%)	3 (1.5%)	0.703*****
Chronic obstructive pulmonary disease	3 (0.6%)	5 (2.5%)	0.059*****
Dyslipidemia	120 (25.8%)	55 (27.2%)	0.690**
Screening and risk assessment tools			
BMI (M-SD)	29.5 (5.2)	29.1 (5.3)	0.411****
MFI (M-SD)	0.10 (0.11)	0.13 (0.10)	<0.001****
SAPS 3 (probability of death) (M-SD)	13.4 (12.3)	24.8 (16.8)	<0.001****
ICU admission SOFA (M-SD)	2.4 (2.9)	5.3 (3.3)	<0.001****
Nutritional risk	183 (40.7%)	100 (49.8%)	0.031**
NRS 2002 (with risk)			
Braden Scale on admission (high risk)	41 (9.7%)	64 (35.8%)	0.001****
Intensive support			
High flow nasal catheter	352 (75.5%)	156 (77.2%)	0.638**
Mechanical ventilation	173 (37.1%)	183 (90.6%)	<0.001**
Non-invasive ventilation	330 (70.8%)	157 (77.7%)	0.065**
Sedation	180 (38.6%)	185 (91.6%)	<0.001**
Prone	40 (8.6%)	67 (33.2%)	<0.001**
Neuromuscular blocker	135 (29%)	159 (78.1%)	<0.001**
ECMO	6 (1.3%)	21 (10.4%)	<0.001**
CRRT	12 (2.6%)	47 (23.3%)	<0.001**
Hospital admission outcome			
Hospital days admitted to the ICU (M-SD)	8.3 (8.2)	25.6 (18.9)	<0.001****
Total hospital admission days (M-SD)	22.3 (22.2)	47.3 (34)	<0.001****
Outcome in the ICU (death)	14 (3%)	47 (23.3%)	<0.001**

M - mean; SD - standard deviation; ICU - Intensive Care Unit; BMI - Body Mass Index; MFI - Modified Frailty Index; SOFA - Sequential Sepsis-related Organ Failure Assessment; ECMO - extracorporeal membrane oxygenation; CRRT - continuous renal replacement therapy; *Student’s t for independent samples; **Pearson’s chi-square; ***Extension of Fisher’s exact test; ****Mann-Whitney; *****Fisher’s exact.

**Table 2:** Risk factors for pressure injuries according to logistic regression analysis. São Paulo, São Paulo, Brazil, 2021

Variable	p value after logistic regression	Odds Ratio	IC 95%
Clinical profile			
Age in years	**<0.001**	1.06	1.03 – 1.08
Diabetes Mellitus	0.005	2.07	1.24 – 3.43
Immunosuppression	0.034	3.40	1.10 – 10.55
Screening and risk assessment tools			
Nutritional risk - NRS 2002	0.015	1.99	1.14 – 3.48
Risk score for PI on admission (reference is high risk)			
No risk	0.039	0.43	0.19 – 0.96
Low risk	0.002	0.36	0.19 – 0.68
Moderate risk	0.326	0.68	0.32 – 1.46
Intensive support			
Mechanical ventilation	**<0.001**	3.52	1.80 – 6.88
Hospital admission outcome			
Hospital days admitted to the ICU	**<0.001**	1.11	1.07 – 1.14

*CI - Confidence Interval; PI - pressure injury; ICU - Intensive Care Unit.*

Mechanical ventilation (MV) was the main predictor of PI, followed by immunosuppression, Diabetes Mellitus, nutritional risk, days of hospital admission to the ICU and age. Patients classified as high risk by the Braden Scale also have a greater chance of developing PI.

## DISCUSSION

The emergence of one of the greatest pandemics in modern history, resulting from COVID-19, has shed light on issues related to PI development in critically ill patients, due to the observation of a likely increase in the occurrence of these injuries^(7)^. This fact may have as its root cause factors that permeate the initial lack of knowledge about the pathophysiology of the new virus and its systemic impacts, such as increased complexity and criticality of patients, in addition to factors related to the health crisis resulting from increased demand for intensive care, such as a shortage of human and material resources within health organizations, in addition to the overload of health professionals, social isolation and many other problems triggered by the emergence of the disease^(9,18,19)^.

This study confirmed the above observation as it identified a high cumulative incidence rate of PI in critically ill patients diagnosed with COVID-19, mainly affecting male patients, older adults and those with other chronic diseases.

Although the pandemic resulting from the new coronavirus has provided extensive discussion about PI and MDRPI occurrence on the world stage, much of the literature produced analyzes specific aspects within this population, such as PI occurrence in patients submitted to prone decubitus^(7,9,20,21)^, differential diagnosis between PI and cutaneous manifestations of COVID-19^(7,8,21,22)^, challenges for preventing PI and MDRPI in the face of the pandemic^(9,10,11,19)^. Therefore, it is not yet possible to elucidate the impact of the virus on PI incidence.

However, when comparing the incidence identified in our study, an increase in PI occurrence can be observed, since data from a systematic literature review, which analyzed PI incidence in critically ill patients prior to the COVID-19 pandemic, showed that rates ranged from 9.4 to 27.5%^(23)^. In agreement with the findings, a national multicenter study also identified a lower incidence (18.7%)^(24)^.

A reasonable justification for the large volume of PI identified in this study refers to the SARS-CoV-2 infection itself, in which the pathophysiology confers additional risk factors for developing PI, with emphasis on systemic coagulopathy, hyper catabolic state and tendency towards greater criticality and hemodynamic instability. Furthermore, use of multiple invasive devices, increased ICU length of stay, prone or elevated decubitus positioning, intolerance to repositioning and organizational factors make PI and MDRPI prevention in these patients even more challenging^(7)^.

If, on the one hand, we saw in this study the frequency of PI occurrence in critically ill patients changed with the advent of COVID-19, on the other hand, we identified similarities regarding PI classification when comparing them with the literature^(25,26,27)^, with a predominance of less severe PI, stages 1 and 2, followed by PI on mucous membrane, with this classification generally linked to the use of devices. That said, in our study we found a relevant volume of PIs resulting from the use of health care devices, corresponding to around 1/3 of all PIs.

Although MDRPI incidence prior to the COVID-19 pandemic in critically ill patients varied greatly (0.9% to 41.2%), there were already estimates that 20 to 40% of all PIs developed in the ICU would have devices as causal factor, also with findings similar to those found in the present study^(28,29)^. Specifically in patients with COVID-19, the literature states that skin damage, resulting from pressure, friction and shear caused by the use of medical devices associated with a cytokine storm, hypercoagulation and hypoxia promoted by the infection, can feed back into the MDRPI development cycle, contributing to an increase in its occurrence^(11)^.

Considering the complexity and particularities of critically ill patients as well as their multiple risk factors for developing PI, Jill Cox and Marilyn Schallom (2021)^(30)^ propose a conceptual scheme, specific to this population. Risk factors are divided into three groups: static intrinsic (age, impaired mobility, smoking, peripheral arterial disease, coronary artery disease, Diabetes Mellitus and severe kidney disease); intrinsic dynamic (include hypotension, respiratory failure, hemodynamic instability, protein-calorie malnutrition and anemia); and dynamic extrinsic factors (length of stay in the ICU, prolonged surgical length and factors related to treatment, such as administration of vasopressors and MV). The scheme suggests the combined assessment of risk factors with tissue tolerance, blood oxygenation/perfusion and pressure, friction, shear forces as well as changes in the microclimate to which patients are subjected.

Having mentioned the multiple risk factors recognized by the scientific community, it is interesting to note that the factors identified in our study, through logistic regression analysis, which presented p<0.05 were, for the most part, encompassed in the conceptual scheme above, as use of MV, immunosup-pression, Diabetes Mellitus, nutritional risk, days spent in ICU and advanced age.

The main risk factor identified in the studied population was the use of MV, with more than 90% of patients who developed PI requiring this therapy during their ICU stay. MV is the most used life support technique in critically ill patients around the world^(31)^, widely used in the management of patients with severe symptoms of COVID-19^(6)^. This use caused worldwide commotion as the scarcity of ventilators available, given the number of affected patients and the prolonged use of MV, strained the entire global healthcare system, bringing healthcare organizations to the brink of collapse^(32)^.

However, even though it is of fundamental relevance for critical patients, especially during a pandemic, MV is not without risks, especially when used for a prolonged period of time^(31)^. Regarding PI development, MV is often described as a risk factor for PI development^(23,24,27,33)^. Its use is often linked to the administration of sedatives and neuromuscular blockers, with a consequent reduction in physical mobility and sensory perception, which may be one reason, among many others, for this association^(1,31)^.

In this study, immunosuppression was also identified as a risk factor for developing PI. This fact is associated with the pathogenesis of the injury itself, given that, when faced with mechanical forces, tissues can suffer ischemia and reperfusion events, lymphatic channel obstruction and cellular deformation, which, in combination or alone, result in elevated inflammation, release of reactive oxygen species and apoptosis, which contribute to dysregulation of the immune response and impaired healing^(34)^.

Another risk factor identified was Diabetes Mellitus, which corroborates the results found in international studies, in which three meta-analyses indicate an association between diabetes and a greater chance of developing PI in surgical patients, varying the odds ratio by 2.15^(35)^, 1.74^(36)^ and 1.77^(37)^.

In the intensive care setting, severity and instability are crucial points for patients’ outcome, and may also influence PI development. It is known that patients diagnosed with COVID-19 had prolonged ICU stays, mainly due to the complexity of the disease and the clinical and hemodynamic repercussions resulting from the infection. In our study, it was observed that, for each day of ICU stay, there is a 1.1-fold increase in patients’ chance of developing PI. Similar studies confirm this association^(24,25)^.

The length of stay is also associated with a greater susceptibility of patients developing malnutrition. It is known that nutrition plays a fundamental role in the capacity for regeneration, absorption of nutrients by the skin and support structures^(38,39,40)^. In the current study, the sample was screened using the NRS (2002), identifying a predominance of nutritional risk in patients who developed PI. It is known that voluntary or involuntary weight loss (nutritional risk evidenced by nutritional screening and tracking), inadequate dietary intake and altered BMI directly impact the risk of PI^(41,42,43)^.

Furthermore, age is another important aspect to be considered regarding PI development. The chance of patients developing PI increased by 1.06 times per year of age. In a national study, the odds ratio increased by 2.3 times in patients aged between 60-84^(24)^. It is known that aging is directly related to a reduction in skin elasticity, in addition to changes in texture, circulation and skin hydration, and a reduction in peripheral sensitivity^(44,45)^. Age is also related to greater risks of systemic complications, especially with regard to elderly patients affected by COVID-19^(46)^.

Faced with so many risks related to PI development in patients admitted to hospital, it is recommended to apply a risk assessment scale^(1)^, appropriate to the patient profile, in order to assist in prevention measure implementation. In the institution where the study was developed, the Braden Scale was used, applied daily by the nurse responsible for the patient. In our results, we identified that patients classified upon admission to the ICU as no risk and low risk by the Braden Scale were less likely to develop PI, when compared to those at high risk of PI. It is known that the Braden Scale, although widely used in Brazilian ICUs, is not specific for use in critically ill patients. Data from a systematic review with meta-analysis, published in 2020, demonstrated that the Braden Scale had moderate predictive validity with good sensitivity, but low specificity for critically ill adult patients^(47)^. Therefore, in addition to applying the Braden Scale, patient clinical assessment is essential, especially in the intensive care setting.

It is worth noting that, in the ICU studied, after identifying some degree of risk using the Braden Scale, a PI prevention bundle is implemented, consisting of multimodal interventions, according to NPIAP 2019 recommendations^(1)^, which include: general skin care; repositioning; moisture management; use of multilayer foam coverings in the sacral region and heels in high-risk patients; specific covers and fasteners to protect device areas; nutritional assessment and intervention; and support surface with viscoelastic foam or with alternating pressure (available for some beds).

Although such preventive measures were already routine at the institution in question, it is worth noting that, during the COVID-19 pandemic, the implementation of one or more prevention interventions may have been limited, since health services, in general, were overwhelmed by the complexity and severity of patients affected by SARS-CoV-2, which is believed to have contributed to the high PI incidence in the population in question.

### Study limitations

The results of this work present limitations in relation to the study model, since retrospective studies depend on the team’s record of PI development. Although researchers have searched for more than one source of information, some data may not have been covered in these searches. However, the sample consisted of medical records of patients admitted to the ICU of a single hospital center.

Given the complexity and particularity of patients infected by COVID-19 on the global stage, more studies are needed to delve deeper into the relationships between intrinsic and extrinsic factors, implementation of preventive interventions and PI incidence.

### Contributions to nursing and health

This study brought data relating to one of the most reported adverse events in recent years, in addition to the COVID-19 pandemic, which directly impacted health services and nursing’s and multidisciplinary team’s performance as a whole. Such findings can support the practice of care, directing the application of best prevention practices and supporting analyzes of the impact of SARS-CoV-2 on PI incidence in patients admitted to the ICU.

## CONCLUSIONS

Accumulated PI incidence in critically ill patients with COVID-19 found in this study was high, 30.2%, mainly affecting individuals who used MV, immunosuppressed, with Diabetes Mellitus, those with nutritional risk upon admission, with prolonged hospital admission in intensive care and advanced age.

It is believed that the difficulties faced by health services in terms of downsizing, supplies as well as overload and stress for professionals who provide direct assistance to these patients, may have influenced the implementation of PI prevention measures during the pandemic period. Linked to this, the severity of the disease combined with the lack of timely scientific information has made care for this type of patient even more complex, and such issues seem to have contributed to increased PI incidence.
